# Roadmap for electrocaloric films characterization

**DOI:** 10.1016/j.isci.2026.115965

**Published:** 2026-05-22

**Authors:** Victor Regis, Urban Tomc, Andrej Kitanovski, Hana Uršič

**Affiliations:** 1 Electronic Ceramics Department, Jožef Stefan Institute, Jamova cesta 39, 1000 Ljubljana, Slovenia; 2 Jožef Stefan International Postgraduate School, Jamova cesta 39, 1000 Ljubljana, Slovenia; 3 Faculty of Mechanical Engineering, University of Ljubljana, Aškerceva cesta 6, 1000 Ljubljana, Slovenia

**Keywords:** simulation in materials science, materials characterization techniques, films

## Abstract

The development of electrocaloric (EC) elements for cooling technologies has progressed by integrating the EC layers onto various substrates. However, a precise understanding of heat losses on direct characterization methods is still lacking, particularly in the infrared (IR) camera method. In this work, we perform numerical simulations on EC film structures to benchmark the substrate influence on the cooling output of the structure for characterization purposes. Substrates with low thermal effusivity (≤3 kW s^1/2^ m^−2^ K^−1^) exhibit minimal influence on the output of films. Simulations were also performed to investigate the impact of EC film and black-paint coating thicknesses on the correction factor for IR characterization methods. A single-digit correction factor can be achieved if the EC film is thicker than the coating. Our results offer a roadmap for designing EC structures for cooling at the micro-to-nano scales.

## Highlights

• Thick-film structures were simulated targeting maximized caloric response
• Substrates with low thermal effusivity (≤3 kW s^1/2^ m^−2^ K^−1^) are suitable
• Substrates with low thermal conductivity (≤1 W K^−1^ m^−1^) are suitable
• Numerical simulations for infrared-based caloric measurements were performed

## Graphical abstract

**Figure:**
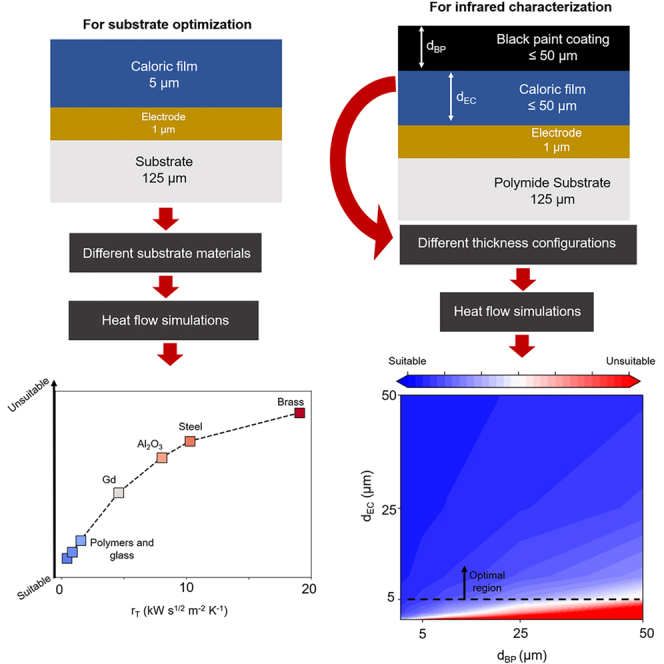

## Introduction

Vapor compression cooling technologies, although widespread and well-established, have a limited Carnot efficiency and do not offer a viable way to be miniaturized at micro or nano scales. In the last few decades, research on cooling alternatives has intensified, particularly on the materials that exhibit caloric effects, which are the adiabatic and reversible temperature change (ΔT_AD_) as a response to external stimuli. Such materials are categorized into electrocalorics (ECs), magnetocalorics, or mechanocalorics, which are responsive to electric field, magnetic field, or mechanical load, respectively.^1^^,^^2^^,^^3^^,^^4^ Particularly, ECs show a promising future for miniaturized cooling devices, as these only require the material layers, electrodes, and the voltage supply, all of which are already present in electronic devices.^4^ The most prominent ECs are Pb-based oxides, such as Pb(Mg_1/3_Nb_2/3_)O_3_-xPbTiO_3_ and Pb(Sc_0.5_Ta_0.5_)O_3_.^5^^,^^6^^,^^7^^,^^8^^,^^9^^,^^10^^,^^11^^,^^12^^,^^13^ In particular, 0.9Pb(Mg_1/3_Nb_2/3_)O_3_-0.1PbTiO_3_ (PMN-10PT) has been extensively studied for its thermal properties as well as its excellent EC performance in film^8^ and ceramic^13^^,^^14^^,^^15^^,^^16^ forms.

EC films are promising for micro-cooling applications as they have been shown to achieve large ΔT_AD_,^4^^,^^5^^,^^6^^,^^7^^,^^8^ and can be integrated onto a variety of substrates: polymers,^7^^,^^8^^,^^9^ metals,^17^^,^^18^^,^^19^^,^^20^ glasses,^19^^,^^20^^,^^21^ and ceramics.^21^^,^^22^^,^^23^ Caloric-based prototypes require excellent thermal properties in order to achieve optimal performance.^24^^,^^25^^,^^26^^,^^27^^,^^28^ However, for characterization purposes, low thermal transport properties are required, otherwise the substrate will absorb all the caloric output, hindering the measurement. In other words, for a reliable quantification and understanding of the intrinsic properties of caloric materials, it is necessary to minimize systematic heat losses, therefore maximizing the measurement response.^8^^,^^29^ In light of this, in recent years, research has focused on finding and optimizing substrates that allow for maximized measurement responses. Despite that, most reports which discuss heat sink effects for characterization purposes consider only case-specific parameters and, to the authors’ knowledge, there is no study on the impact of substrate thermal parameters, such as heat capacity (c_p_), thermal conductivity (λ), and density (ρ), on the effective cooling output of caloric film structures, particularly for characterization purposes.

Characterization methods of the caloric response of materials are generally classified as either direct or indirect. The indirect approach offers a benchmark ΔT_AD_ value, however it does not take into consideration effects such as Joule heating and heat losses to the substrate, resulting in potentially inaccurate values.^8^ To circumvent such an issue, direct characterization methods can be performed. A common drawback of direct methods is the heat losses related to the measurement setup; in other words, the intrinsic ΔT_AD_ is proportional to the measured temperature change (ΔT_M_) by a constant k, called the correction factor. Mathematically, it can be described as ΔT_AD_ = k ΔT_M_, where k can be determined by assessing method-specific experimental parameters. For example, the thermistor-in-calorimeter method requires wiring, a bead-thermistor, glue and has an acquisition time of ∼1 s, which leads to a factor of 30–50.^8^^,^^9^ The correction factor can be significantly reduced by means of a non-contact infrared (IR) characterization method, which has been typically used for magnetocaloric and elastocaloric characterizations.^30^^,^^31^^,^^32^^,^^33^^,^^34^^,^^35^^,^^36^^,^^37^ Using the IR method, only a thin layer of a high-emissivity coating, generally black paint, is required, and the acquisition time may be as fast as ∼1 ms.^29^^,^^30^^,^^31^ It has been shown that the IR characterization method can also be used in EC thick films. With the method, single-digit k-values have been achieved on μm-thick EC films prepared on polymer substrates.^7^^,^^8^^,^^29^

In recent years, the IR method has become more widespread, with a wide range of samples being characterized. However, most studies do not report the thickness of the paint, and many do not consider the influence of the black coating on the measurements, likely assuming that the correction factor is negligible.^38^^,^^39^^,^^40^ In other studies, targeting the direct characterization of EC μm-thick films, the EC film thickness (d_EC_) and black paint coating (d_BP_) are both considered and simulated, albeit only using case-specific parameters.^8^^,^^29^ Although the literature reports consistent and accurate results, the influence of d_EC_ and d_BP_ on the correction factor remains unaddressed.

In light of this, in this work, using a finite-element model, we numerically investigate the influence of the substrate thermal parameters on the cooling performance of a caloric film structure, aiming to optimize measurement response. Due to its excellent properties, PMN-10PT was chosen as the reference EC material. For the IR method, the impact of the d_EC_, as well as black coating thicknesses, d_BP_, on the correction factor k was investigated. This work aims to establish a preliminary assessment on substrate material as well as benchmark sample-to-coating optimization for the IR characterization method of caloric samples.

## Results and discussion

### Numerical model of electrocaloric structures

**Figure 1:**
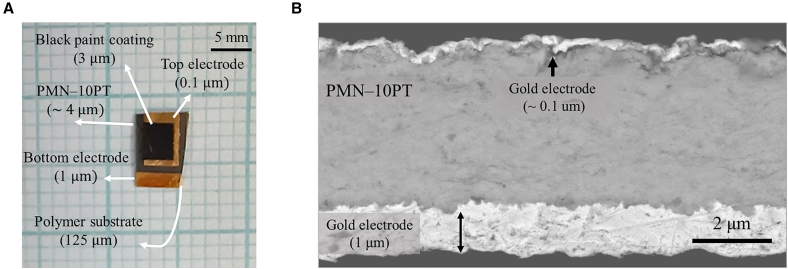
Photograph and microstructure images of the reference sample (A) Photograph of PMN-10PT thick film on gold-sputtered polymer substrate covered with gold top electrode and coated with black paint layer for IR characterization. The scale bar represents 5 mm. (B) SEM image of a sample in cross-sectional view. The scale bar represents 2 μm. Note the large thickness difference between the top and bottom electrodes.

A typical EC thick-film sample, prepared via the aerosol deposition method, coated for IR characterizations^7^^,^^8^ is shown in Figure 1A. Generally, for EC measurements, it is desired to have electrodes as thin as possible to minimize substrate influence. However, in the case of aerosol-deposited samples, due to processing constraints, the bottom electrode must be at least 1 μm-thick to ensure proper film adhesion.^8^^,^^22^ Such a structure was used as a reference geometry for the simulations. A scanning electron microscopy image of a sample in a cross-sectional view is shown in Figure 1B.

**Figure 2:**
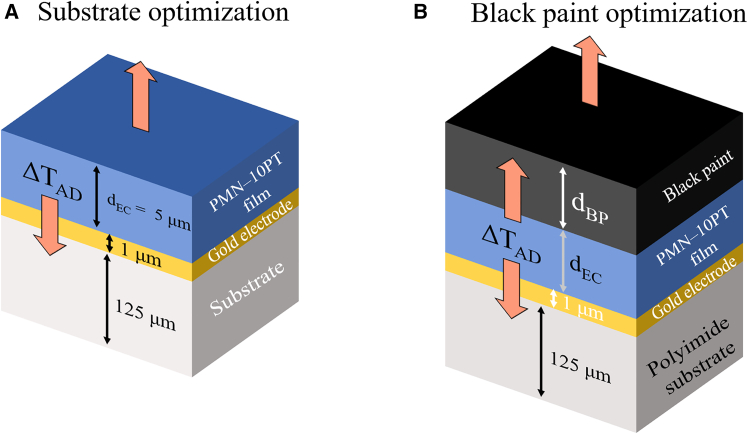
Simulated structures for substrate material and black paint optimizations Schematic representation of simulated structures for (A) substrate optimization and (B) correction factor optimization. The arrows indicate the heat flow direction after the EC pulse is active.

The investigations were performed via a 2-dimensional finite-element model using COMSOL multiphysics within the interval 0 s to 50 ms at 10 μs steps. The simulations were then split into two segments: minimizing the influence of the substrate and minimizing the correction factor for IR camera measurements. For the former, d_EC_ was fixed at 5 μm, and different values of ρ, cp, and λ were investigated. For the latter, an additional d_BP_ atop of the previous structure was implemented, and polyimide was set as the substrate material. Structures with different combinations of d_EC_ and d_BP_ were simulated, with both values within 0.5 μm and 50 μm. The simulated structures are schematically shown in Figure 2. The EC effect was implemented through the power density, P_EC_, described as follows^8^^,^^29^:

(Equation 1) $$P_{\text{EC}}=\left\{ \begin{array}{c} 0,t<10\;\mu s\\ \frac{\rho C_{p}\Delta T_{\text{AD}}}{\tau },10\;\mu s\leq t\leq \tau \\ 0,t\;>\;\tau_{\text{pul}} \end{array}\right.$$

where τ is the duration of the caloric pulse, and it was set to 1 ms to simulate a fast thermal exchange rate.

It should be noted that the intrinsic ΔT_AD_ is proportional to the ΔT_s_, which reaches the surface layer of the system. For the substrate optimization, the proportionality constant, δ, quantifies only the losses to the substrate, henceforth called the heat sink coefficient, defined as:

(Equation 2) $${\Delta T}_{AD}={\delta \Delta T}_{S}$$

For IR camera optimizations, the black paint further contributes to the losses. In this case, the intrinsic ΔT_AD_ is proportional to the measured ΔT_M_ by a constant k, which is an experimental parameter conventionally called the correction factor, mathematically described as:

(Equation 3) $${\Delta T}_{AD}={k\Delta T}_{M}$$

For the simulations, PMN-10PT was used as the reference material due to its excellent ΔT_AD_^8^^,^^16^ as well as moderate thermal properties.^14^ To accurately represent the influence of the black paint, coatings were prepared using the airbrush method^41^ and the black paint c_p_ was characterized, a value of 1350 J kg^−1^ K^−1^ was measured at room temperature, shown in supplemental information S1. The numerical values of the physical parameters of PMN-10PT, gold, and black paint used in the simulations are shown in Table 1.

**Table 1:** Physical parameters of materials used in the simulations

Parameter	Electrocaloric PMN-10PT^8^^,^^14^	Gold electrode^8^^,^^29^	Black paint^8^^,^^42^
c_p_ (J kg^−1^ K^−1^)	349	129	1350^S1^
ρ (kg m^−3^)	8120	19300	1160
λ (W m^−1^ K^−1^)	1.3	318	0.2
ΔT_AD_	1 K	–	–
τ	1 ms	–	–

### Verification of the numerical model

**Figure 3:**
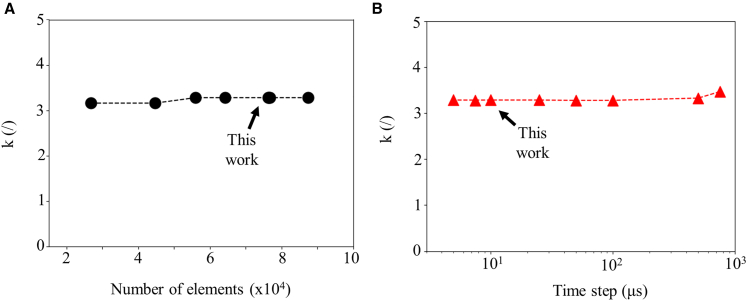
Proof of convergence of simulations Dependence of the correction factor on (A) the number of elements and (B) the time resolution.

The EC structure model was verified for its numerical consistency by checking the sufficiency of spatial and temporal discretization. For the validation, the structure for IR characterization was selected; in other words, the substrate was set to polyimide, and the black paint layer was considered. In particular, d_EC_ and d_BP_ were set to 5 μm. As shown in Figure 3A, a number of elements larger than 5 ×10^4^ is needed for the simulations to converge. Figure 3B shows k values as a function of different temporal resolutions, with 100 μs being the largest convergent value. Hence, to ensure convergence across all cases, in this work, 7.5 ×10^4^ elements were used and a time resolution of 10 μs was implemented. In addition, the calculated Knudsen number of the system was much smaller than 0.01, indicating that the system is in the continuum regime, therefore within converging limits, satisfying the conditions for the heat flow model.^43^^,^^44^^,^^45^^,^^46^

#### Minimizing the heat sink contribution from the substrate

The heat sink coefficient, δ, quantifies the thermal influence of the substrate material on the cooling performance of the caloric structure, as defined in Equation 2. If there is no heat transfer between the caloric film and the substrate, δ achieves its minimum value of 1. Therefore, the substrate materials that lead to δ as close to 1 as possible are the most suitable. In other words, to evaluate the influence of a substrate material on δ, it is necessary to quantify the ability of the substrate to transfer heat to its surroundings, namely, its thermal effusivity (r_T_), mathematically described as^42^^,^^47^:

(Equation 4) $$r_{T}=\sqrt{c_{p}\lambda \rho }$$

**Figure 4:**
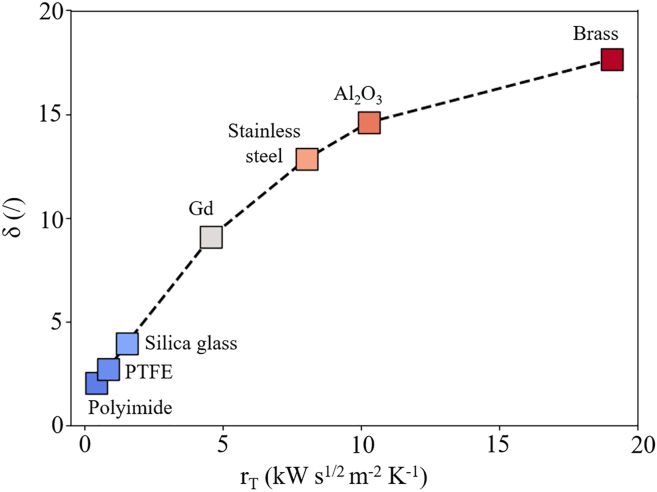
Heat sink coefficient as a function of thermal effusivity Heat sink coefficient δ as a function of r_T_ for different materials. Blue markers represent low δ, while red markers represent higher δ values.

In the simulations, a wide range of materials was implemented as substrates, namely polymers, metals, silica glass and ceramic Al_2_O_3_. As shown Figure 4, substrate materials that exhibit effusivity r_T_ ≤ 3 kW s^1/2^ m^−2^ K^−1^, such as polyimide, polytetrafluoroethylene (PTFE) and silica glass, are the most suitable as such structures can achieve the lowest single-digit δ due to overall poor thermal exchange properties of the substrates. Whereas materials such as Gd, stainless steel, and Al_2_O_3_, exhibit 9 < δ < 15, in other words, these materials are potentially suitable for the substrates, depending on specific physical parameters of the system and experimental conditions. Among the simulated materials, brass exhibits the largest value of δ ∼18. The c_p_, λ, ρ, r_T_ of the substrate materials and the corresponding δ of the simulated EC structures are collected in Table 2.

**Table 2:** c_p_, λ, and ρ for different substrate materials at 300 K

Material	c_p_ (J kg^−1^ K^−1^)	λ (W m^−1^ K^−1^)	ρ (kg m^−3^)	rT (kW s^1/2^ m^-2^ K^−1^)	δ (/)
Polyimide^8^^,^^42^	904	0.1	1420	0.36	2.0
PTFE^48^^,^^49^^,^^50^	1100	0.3	2200	0.85	2.7
Glass^51^^,^^52^	750	1.4	2200	1.51	3.9
Gd^53^^,^^54^	300	8.8	7900	4.57	9.1
Stainless steel^42^	500	16.2	7930	8.01	12.8
Al_2_O_3_^55^^,^^56^^,^^57^	880	30	3990	10.26	14.6
Brass^42^^,^^58^^,^^59^	380	111	8600	19.05	17.7

The simulated r_T_ and δ are also shown.

To further assess the substrate influence, the individual contributions of c_p_, λ, and ρ of the substrate on the δ values of the systems were also investigated. Using a simulated structure shown in Figure 1A, multiple combinations of the thermal parameter values were investigated. The selected c_p_, λ, and ρ values were chosen to represent a wide range of materials for substrates, including polymers, ceramics, and metals.

**Figure 5:**
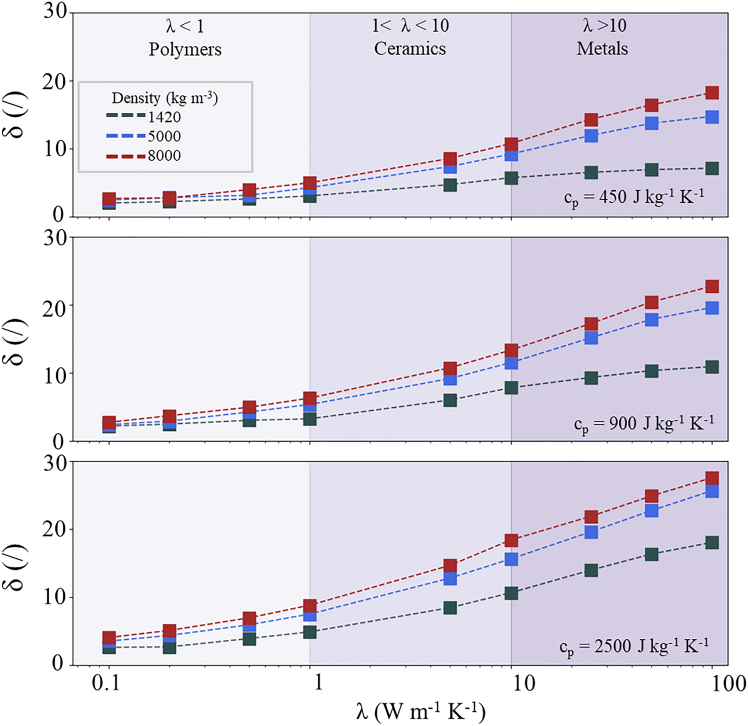
Heat sink coefficient as a function of different thermal parameters Heat sink coefficient δ as a function of thermal conductivity at different substrate c_p_ values: 450 J kg^−1^ K^−1^ (upper panel), 900 J kg^−1^ K^−1^ (middle panel), 2500 J kg^−1^ K^−1^ (lower panel). The colored regions within the panels represent the thermal conductivity range of polymers (λ < 1 W m^−1^ K^−1^), ceramics (1 W m^−1^ K^−1^ ≤ λ ≤ 10 W m^−1^ K^−1^), and metals (λ > 10 W m^−1^ K^−1^). The marker colors represent different densities. The dashed lines are a guide to the eyes.

In the first numerical analysis, c_p_ of the substrate was set to 450 J kg^−1^ K^−1^ (Figure 5, upper panel). These values approximately correspond to various metals and ceramics. Albeit at low λ, a comparatively low δ can be obtained, metals tend to exhibit larger density compared to ceramics and polymers and large λ, resulting in larger heat sink effects, reaching δ ∼20. Then, c_p_ was set to 900 J kg^−1^ K^−1^, representing ceramics and polymers, shown in the middle panel of Figure 5. Particularly, polyimide with λ = 0.2 W m^−1^ K^−1^ and ρ = 1420 kg m^−3^ results in δ = 2. In contrast, Al_2_O_3_ shows a larger thermal conductivity, λ = 30 W m^−1^ K^−1^ and larger density ρ = 3990 kg m^−3^, leading to δ ∼15. Lastly, materials with large c_p_ (2500 J kg^−1^ K^−1^) were also simulated, shown in the bottom panel of Figure 5. At large ρ and λ, the coefficient δ can reach values as large as 30. However, at low ρ and λ, for example, in the case of polyethylene that exhibits low density (ρ < 1420 kg m^−3^) and low thermal conductivity (λ < 0.5 W m^−1^ K^−1^),^42^^,^^60^ the heat sink coefficient results in δ < 4. Notice that in all panels, single-digit δ can be achieved regardless of c_p_ and ρ, if λ ≤ 1 W m^−1^ K^−1^. In other words, a low thermal conductivity of the substrate is a sufficient condition to minimize thermal losses.

#### Minimizing the correction factor in IR characterizations

**Figure 6:**
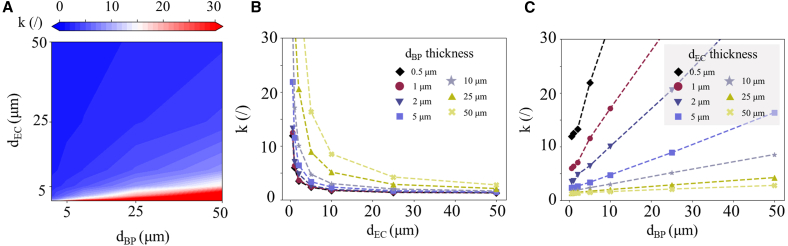
Correction factor as a function of electrocaloric film thickness and black paint thickness (A) 2D mapping of the correction factor as a function of d_EC_ and d_BP_. Correction factor k as a function of (B) d_EC_, and (C) d_BP_.

The influence of the d_EC_ and the d_BP_ thickness on the correction factor in IR characterizations was investigated. Polyimide (ρ = 1420 kg m^−3^, c_p_ = 904 J kg^−1^ K^−1^, λ = 0.2 W m^−1^ K^−1^)^8^ was used as a substrate material due to its minimal δ contribution. Simulations were performed with different d_EC_ and d_BP_ combinations, from sub-micron sizes up to 50 μm, as shown in Figure 2B. The values of k as a function of both d_EC_ and d_BP_ are shown in Figure 6A. Notice that regions of k ≤ 10 (blue regions) are more predominant than regions of k ≥ 20 (red regions), suggesting that increasing d_EC_ plays a more important role in minimizing k than decreasing d_BP_. Particularly, most of k ≤ 10 is found above the threshold d_EC_ ≥ 5 μm, suggesting that k is strongly dependent on d_EC_.

Figures 6B and 6C show k as a function of d_EC_ and d_BP_, respectively. Notice that while k decreases nonlinearly with d_EC_, it increases linearly with d_BP_. It should be emphasized that d_BP_ can be as thin as a few μm, while larger thicknesses can be prepared with additional coatings.^41^ Assuming d_BP_ ≤ 40 μm, in order to achieve single-digit k values, at least one of the following conditions must be satisfied: d_EC_ ≥ d_BP_ or d_EC_ ≥ 5 μm. In bulk materials, d_EC_ ≫d_BP_, as the samples are millimeter-thick, while coatings do not exceed the micrometer-range, leading to a negligible correction factor. Thin films, however, generally have d_EC_ ≤ 0.5 μm, and the expected correction factor should be considerably large, k ≫ 30, hindering the IR characterization method as a viable option. In the case of thick-film characterizations, both d_EC_ and d_BP_ are a few micrometers thick, requiring case-specific optimizations in order to obtain k ≤ 10. In addition, the case of free-standing films is discussed in supplemental information S2. Our results are in good agreement with the literature, where a 3-μm-thick PMN-10PT sample with a 3.5-μm-thick coating was estimated to have a k∼3.4 on a polyimide substrate.^8^ In our simulations, under similar conditions, k ∼3.8 can be obtained. The difference between the values is attributed to the different thermal parameters of the black paints.

It is worth mentioning that in most practical applications, heat loss effects are expected to be larger than those calculated here, as the simulations were performed assuming ideal conditions (thermally insulated, no electrical contacts, low acquisition time), therefore, our values of δ and k represent lower-bound estimates. In other words, structures that exhibit large δ values according to our work will likely exhibit large values in real-world applications. In addition, this work is based on PMN-10PT as the active material, and, evidently, distinctive materials will interact differently and should be considered on a case-by-case basis. Further discussion on the influence of different materials, IR camera frame rate, and systematic contributions can be found in supplemental information S3.

In conclusion, using numerical simulation, we have investigated the substrate influence on the EC cooling output of PMN-10PT thick-film structures. Our results indicate that substrate materials with thermal effusivity r_T_ ≤ 3 kW s^1/2^ m^−2^ K^−1^ lead to minimal heat losses. Particularly, the condition λ_substrate_ ≤ 1 W m^−1^ K^−1^, regardless of c_p_ and ρ, is sufficient to achieve the heat sink coefficient δ ≤ 10, while further minimization can be achieved with substrate materials with lower overall r_T_. In this manner, we have benchmarked the values for the thermal properties of substrates in the EC thick-film structures targeting optimized caloric performance.

In addition, the influence of the EC layer thickness and d_BP_ thickness on the correction factor for the IR characterization method was investigated. Our simulations show that single-digit k can be achieved if at least one of the following conditions is met: if the d_EC_ ≥ 5 μm, assuming d_BP_ ≤ 40 μm; or d_EC_ ≥ d_BP_, with layers thicker than 1 μm. Both conditions are satisfied in the case of bulk materials, while fine-tuning is required for the characterization of thick films. Our results confirm that coating methods that can achieve d_BP_ as thin as possible, such as printing and airbrushing, are highly desirable for IR-based characterization techniques.

This work provides a framework for preliminary assessment on substrate materials as well as sample-to-black coating optimization for the IR characterization method, and offers a roadmap for designing and characterizing caloric film structures for cooling at the micro-to-nano scales.

### Limitations of the study

The main limitation of the work is the ideal case assumption, which results in lower estimates for the heat sink coefficient and correction factor. While it was briefly discussed in the supplemental information, further adjustments and considerations should be made on a case-by-case basis to ensure an accurate estimate of the parameters. In addition, this work focuses on the reference material PMN-10PT, which shows generally intermediate thermal properties. However, caloric effects can be observed on a diverse range of materials, including polymers (thermal insulators) and metals (thermal conductors). In other words, different thermal behaviors can be expected for different caloric materials and should be taken into consideration accordingly.

## Resource availability

### Lead contact

Further information and requests for resources should be directed to and will be fulfilled by the lead contact, Prof. Dr. Hana Uršič (hana.ursic@ijs.si).

### Materials availability

This study did not generate new unique materials.

### Data and code availability

• The data that support the findings of this study are openly available in Zenodo at https://doi.org/10.5281/zenodo.18233562, reference number.^61^
• This study generated no custom code.
• Any additional information required to analyze the data reported in this study is available from the lead contact upon request.

## Acknowledgments

The authors acknowledge the financial support of the Slovenian Research and Innovation Agency (projects no. J2-60035, program no. P2-0105, and young researcher project) and the transnational consortium M-ERA.NET for the project Cool BatMan: Battery Thermal Management System Based on High Power Density Digital Microfluidic Magnetocaloric Cooling (no. 9400, Slovenian part of the project is financed by Ministry of Higher Education, Science and Innovation, Slovenia).

## Author contributions

Conceptualization: H.U. and V.R.; data curation: V.R.; formal analysis: V.R.; funding acquisition: H.U. and U.T.; investigation: H.U., V.R., U.T., and A.K.; methodology: U.T. and V.R.; resources: H.U.; software: H.U. and V.R.; supervision: H.U., U.T., and A.K.; validation: H.U., U.T., and V.R.; writing – original draft: H.U. and V.R.; writing – review and editing: H.U., V.R., U.T., and A.K.

## Declaration of interests

A. Kitanovski is a member of iScience’s Advisory Board and one of the co-guest editors of the special issue “Advanced thermal control: Fundamentals and applications.”

## STAR★Methods

### Key resources table

**Table:** 

REAGENT or RESOURCE	SOURCE	IDENTIFIER
**Deposited data**		

Raw data	This paper	https://doi.org/10.5281/zenodo.18245578

**Software and algorithms**		

COMSOL Multiphysics	COMSOL AB	comsol.com
Python 3	Python Software Foundation	python.org

**Other**		

SEM Verios HP 4G	Thermo Fisher Scientific	thermofisher.com
Acryllic black paint	Vallejo	70.602
DSC 204 F1	Netzsch Group	netzsch.com

### Method details

#### Scanning electron microscopy

The cross-section of the reference sample was analyzed using a scanning electron microscope (SEM; Verios HP 4G, ThermoFisher, USA). Prior to the imaging, the sample was prepared into an epoxy resin matrix and fine polished.

#### Numerical modelling

The heat flow dynamics of the caloric system was simulated via a 2-dimensional finite-element model (COMSOL® Multiphysics version 6.2) using the thermal management module and time-dependent solver, within the interval 0 s to 50 ms at 10 μs steps. In all cases, the substrate had a fixed thickness of 125 μm, and a 1 μm-thick gold bottom electrode, the width of all layers was set to 5 mm. It is worth emphasizing that in the case of aerosol-deposited films, considered the reference structure, the thickness of the top electrode is at most ∼0.1 μm and, therefore, it was not implemented in the simulations. The influence of contacts for voltage application was assumed to be negligible, and all surrounding edges of the system were considered thermally insulated.

### Quantification and statistical analysis

The simulation data is produced by COMSOL Multiphysics. Figures shown in the main text were produced in Python and Microsoft PowerPoint.
