# Supplementary material for: Roadmap for electrocaloric films characterization
Source: iScience. 2026 May 22;29(6):115965. doi: 10.1016/j.isci.2026.115965 (PMC13218226; doi:10.1016/j.isci.2026.115965)
Supplement: Document S1. Figures S1–S4 and Table S1 [file mmc1.pdf]

iScience, Volume *29*

Supplemental information

Roadmap for electrocaloric films characterization

Victor Regis, Urban Tomc, Andrej Kitanovski, and Hana Uršič

Supplemental Information

S1. Heat capacity characterization of black paint

To characterize the heat capacity of the black paint (Vallejo Primer Black, Vallejo, Barcelona, Spain), a black paint layers were deposited via the airbrush method (Filmotool DG-30, Filmotool, Ljubljana, Slovenia) on 76 mm x 26 mm glass substrates (Hirschmann Labogeräte, Eberstadt, Germany) and subsequently removed using a conventional laboratory spatula. The temperature dependence of the C_p was determined with a differential scanning calorimeter (DSC, Netzsch DSC 204 F1, Germany). Measurements were performed on disc-shaped plates (2r ~6 mm, d = 0.125 mm) placed in a Pt crucible with a lid. To exclude possible thermal effects related to evaporation of water and desorption of gasses from the surface of the sample, two heating and cooling cycles were performed. In the first cycle, the samples were heated from 25 °C to 120 °C and then cooled to 0 °C with liquid nitrogen. Subsequently, the samples were heated again to 120 °C and cooled to 25 °C. Heating and cooling rates of 5 K min^{-1} were used for all measurements. Sapphire (Netzsch, 2r = 5.2 mm, d = 0.25 mm) was used as the standard material to determine the C_p of the sample. The Cp measurement is shown in Figure S1.

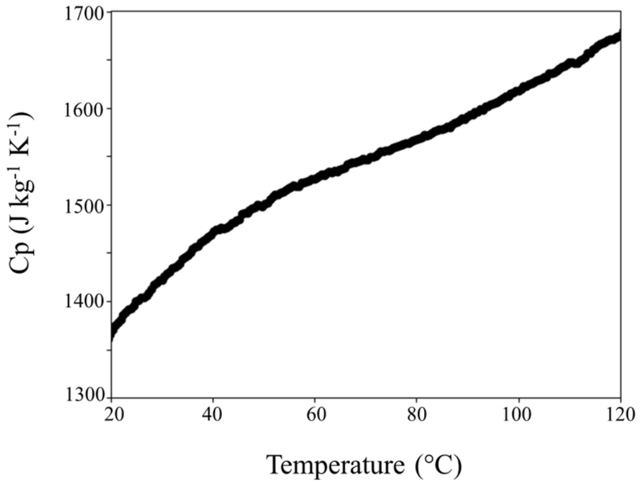

Figure S1: Heat capacity of acrylic-based black paint. Related to Table 1.

S2. Thickness dependence of the correction factor k for free-standing PMN–10PT films

The correction factor k in the case of free-standing films was also considered. The simulations were performed with different thicknesses of free-standing PMN–10PT films. The thickness of the black paint d_{BP} was 5 μm in all cases. As shown in Figure S2, when the thickness of a free-standing PMN–10PT film is 5 μm, a correction factor of ~2 can be obtained. As the thickness of the free-standing PMN–10PT film exceeds that of the black coating, the correction factor converges to

unity, reaching ~1.3 at 50 μm. It is worth mentioning that bulk samples are generally thicker than 200 μm, further decreasing the influence of the black paint.

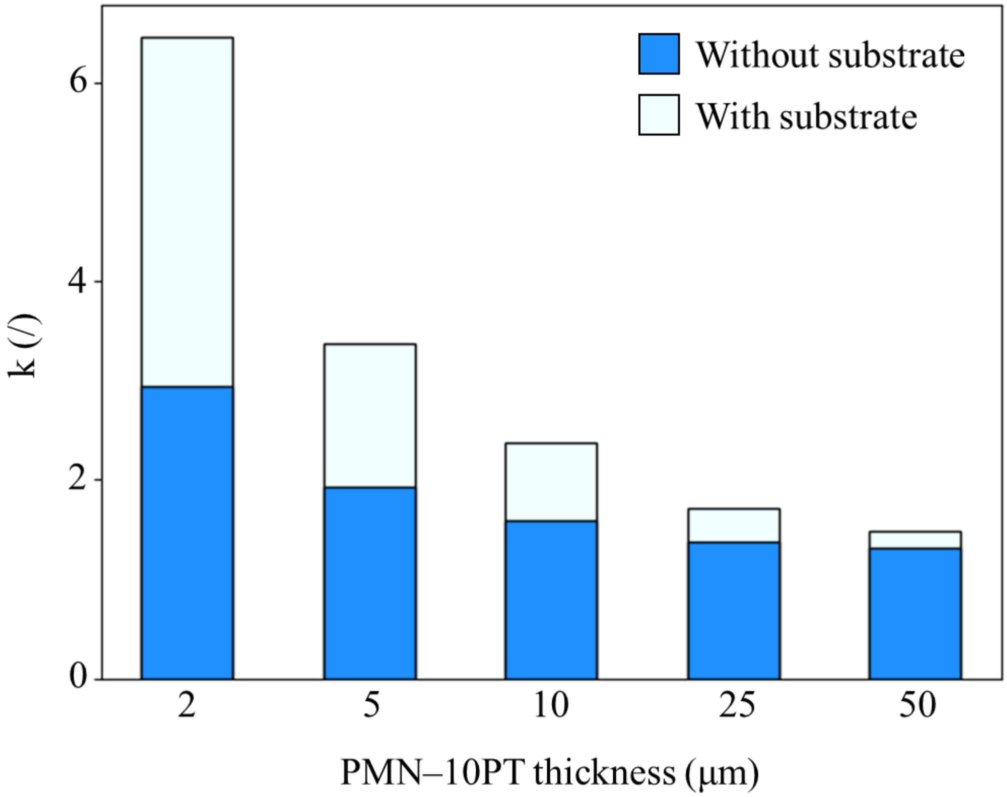

Figure S2: Correction factor as a function of free-standing PMN–10PT thickness. The case considering a polyimide substrate was included for reference. Related to Figure 5.

S3. Parameter dependence of the correction factor k: active material, substrate, and measurement acquisition time

In the main text, we discussed the correction factor on caloric film structures assuming PMN–10PT as the active layer and ideal conditions (thermally insulated, no electrical contacts, low acquisition time), with no additional influence beyond the layers. When assessing different substrates and optimizing the coatings, this approach promptly offers a lower bound estimate for the correction factor. However, real-world infrared-based measurements can be done on a wide range of materials and other parameters may also differ between experimental equipment. In light of this, in this section, the influence of key experimental conditions on the correction factor will be discussed.

First, the impact of different materials as active films was investigated. The simulated materials represent a wide range of thermal properties: P(VDF-TrFE-CFE) is a highly insulating EC polymer (r_T ~0.8 kW $s^{1/2}$ m^{-2} K^{-1})[1], $BaTiO_3$ (BTO) is an EC ceramic material with r_T ~2.6 kW $s^{1/2}$ m^{-2} K^{-1} ,[2]

and Gd is a magnetocaloric (MC) material with comparatively large effusivity, showing r_T ~4.6 kW $s^{1/2}$ m^{-2} K^{-1}.[3,4] Similar criteria on the broad range of thermal properties (as explained in the main text) were used to select the simulated substrate materials: polyimide, glass, and Al_2O_3 substrates. The simulations were performed with the same parameters as the simulations in the main text (Figure 2b). In all the simulations, the thicknesses of films and black paint coatings were set to 5 µm, while the thickness of the substrates was set to 125 µm.

The P(VDF-TrFE-CFE) polymer material exhibits a low thermal effusivity value, and therefore, the P(VDF-TrFE-CFE) films on all three substrates achieve a single-digit correction factor (Figure S3, left group of bars). The ceramic material BTO exhibits single-digit correction factors on polyimide and glass substrates, however, k ~ 20 in the case of Al_2O_3 substrate. Gd also exhibited single-digit correction factors on polyimide and glass substrates, while on Al_2O_3 substrate, k ~ 22.

It is worth emphasizing that thick-film structures for all simulated active materials exhibited comparable k values on both polyimide and glass substrates (orange bars and blue bars in Figure S3). However, this is not the case for Al_2O_3 substrates (grey bars), where the k value changes for different active materials. This is because the Al_2O_3 substrates have the highest thermal effusivity among all three simulated substrates, which imposes significant variance on k values (from 6 to 22). In other words, for Al_2O_3 substrates, a low-effusivity active thick-film material is recommended. The thermal properties of the simulated active materials are collected in Table S1.

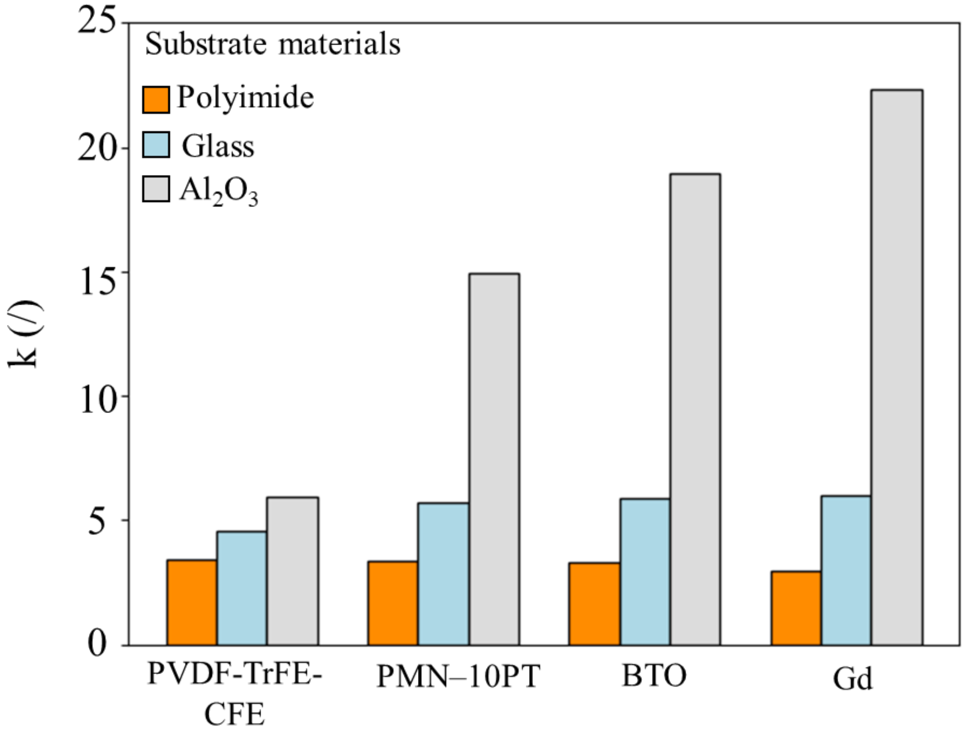

Figure S3: Correction factor, k, of different active thick-film materials on three different substrates. A bottom electrode was not simulated in the case of Gd thick-film structure, as it is not required for MC applications. As a reference, PMN–10PT was also included. Related to Figure 5.

Table S1: Physical parameters of different active materials. Related to Figure 5.

Material	c_p (J kg^{-1} K^{-1})	λ (W m^{-1} K^{-1})	ρ (kg m^{-3})	r_T (kW $s^{1/2}$ m^{-2} K^{-1})
P(VDF-TrFE-CFE)[1]	1780	0.2	1800	0.8
$BaTiO_3$[2]	434	2.7	5900	2.6
Gd[3,4]	300	8.8	7900	4.6
PMN–10PT[5,6]	349	1.3	8120	1.9

Moreover, the acquisition time (inverse of frame rate) of the measurement system plays a significant role in the value of the correction factor. To investigate this influence, simulations were performed with different frame rates, from 10 Hz to 1000 Hz. The simulated structure was the same in all cases, with both the thickness of black paint coating and PMN–10PT layer set to 5 μm, and the thickness of the polyimide substrate set to 125 μm. As shown in Figure S4, at a frame rate of 10 Hz, the correction factor k can reach a value as large as ~ 12.1, which strongly decreases with the frame rate. At 100 Hz, the correction factor k is halved (k ~ 6.2), ultimately decreasing to k ~ 3.4 at 1 kHz.

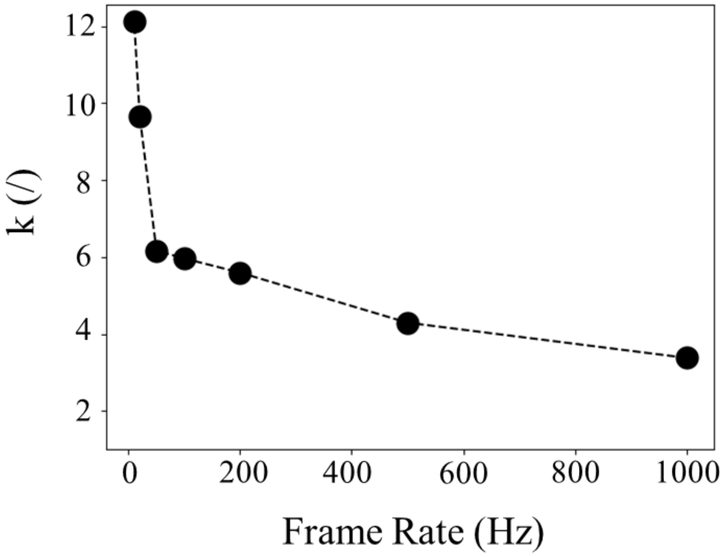

Figure S4: Correction factor, k, as a function of frame rate. Related to Figure 5.

At lower frame rates, there is enough time for the heat to dissipate further into the measurement setup, and, therefore, other systematic contributions should be considered when calculating the correction factor. At such a time scale, the correction can also be obtained via:[6–9]

$$k = \frac{c_p^A}{c_p^T}, \quad (1)$$

where Cp^A and Cp^T are the heat capacity of the active area (under the electrodes) and the total heat capacity of the system, respectively.

While the IR-camera method only requires coating the sample with a black paint layer and can be operated over a wide range of frame rates, the thermistor-in-calorimeter (TiC) method is limited to 1 Hz and additionally requires wiring, a thermistor, and adhesive materials [6–9]. Due to the larger passive thermal masses of the wires, the thermistor, and glues, and the large time scale (∼1 s) of the TiC method, we conclude that $k_{TiC} > k_{IR}$. Within this scope, calculating k_{TiC} offers an upper limit to the systematic contributions of k_{IR}. For the TiC method, a correction factor of ∼27 is obtained for 5-µm-thick PMN–10PT film on a polyimide substrate. In this case, the polyimide substrate contributes with 85 % of this k value, while wiring, glues, and thermistor correspond to the additional 15 % of k value. Since the systematic contributions of the TiC method are always larger than those of the IR camera method, a variance in k_{IR} is in the range of 15 % can be estimated. This value is expected to decrease at larger frame rates, as indicated in Figure S4.

References

1. Aravindhan, A., Lheritier, P., Torelló, A., Prah, U., Nouchokgwe, Y., El Moul, A., Chevalier, X., Domingues Dos Santos, F., Defay, E., and Kovacova, V. (2023). Direct measurement of electrocaloric effect in P(VDF-TrFE-CFE) film using infrared imaging. J. Materiomics *9*, 256–260. https://doi.org/10.1016/j.jmat.2022.10.009.

2. He, Y. (2004). Heat capacity, thermal conductivity, and thermal expansion of barium titanate-based ceramics. Thermochim. Acta *419*, 135–141. https://doi.org/10.1016/j.tca.2004.02.008.

3. Dan’kov, S.Yu., Tishin, A.M., Pecharsky, V.K., and Gschneidner, K.A. (1998). Magnetic phase transitions and the magnetothermal properties of gadolinium. Phys. Rev. B *57*, 3478–3490. https://doi.org/10.1103/PhysRevB.57.3478.

4. Jacobsson, P., and Sundqvist, B. (1989). Thermal conductivity and electrical resistivity of gadolinium as functions of pressure and temperature. Phys. Rev. B *40*, 9541–9551. https://doi.org/10.1103/PhysRevB.40.9541.

5. M. Vrabelj, L. Fulanovič, A. Bradeško, S. Drnovšek, B. Malič, H.U. (2015). Specific Heat Capacity and Thermal Conductivity of the Electrocaloric (1-x)$Pb(Mg_{1/3}Nb_{2/3})O_3$–x$PbTiO_3$ Ceramics Between Room Temperature and 300 °C. Informacije MIDEM 45, 260–265.

6. Prah, U., Sadl, M., Torello, A., Lheritier, P., Kovacova, V., Ursic, H., and Defay, E. (2023). Direct Electrocaloric Characterization of Ceramic Films. Small Methods *7*, 202300212. https://doi.org/10.1002/smtd.202300212.

7. Kutnjak, Z., Rožič, B., and Pirc, R. (2015). Electrocaloric Effect: Theory, Measurements, and Applications. In Wiley Encyclopedia of Electrical and Electronics Engineering, J. G. Webster, ed. (Wiley). https://doi.org/10.1002/047134608X.W8244.

8. Rožič, B., Malič, B., Uršič, H., Holc, J., Kosec, M., Neese, B., Zhang, Q.M., and Kutnjak, Z. (2010). Direct Measurements of the Giant Electrocaloric Effect in Soft and Solid Ferroelectric Materials. Ferroelectrics *405*, 26–31. https://doi.org/10.1080/00150193.2010.482884.

9. Correia, T. (ed.), and Zhang, Q. (ed.) (2014). Electrocaloric Materials: New Generation of Coolers (Springer).
